# Network topology and cannabis use following two weeks of monitored abstinence: moderation of sex and patterns of use findings

**DOI:** 10.3389/fradm.2025.1549771

**Published:** 2025-03-25

**Authors:** Kyle A. Baacke, Ryan M. Sullivan, Chase A. Shankula, Krista M. Lisdahl

**Affiliations:** 1 Department of Psychology, Brain Imaging and Neuropsychology Lab, University of Wisconsin-Milwaukee, Milwaukee, WI, United States,; 2 Department of Psychiatry, Brain Addiction and Development Laboratory, University of California, San Diego, La Jolla, CA, United States

**Keywords:** cannabis, networks, fMRI, young adult, abstinence

## Abstract

**Background:**

Chronic cannabis use (CU) can result in subtle deficits in cognitive performance that may be linked with alterations in underlying neural functioning. However, these network alterations are not well-characterized following monitored abstinence. Here, we evaluate differences in functional brain network activity associated with CU patterns in adolescents/young adults.

**Methods:**

Functional connectomes were generated using resting-state fMRI data collected from 83 healthy young adults (44 male) following two weeks of monitored cannabis abstinence. Network topology metrics were calculated for each of the 7 Yeo 2011 intrinsic connectivity networks (ICNs) and on the whole-brain level. Multiple linear regressions were used to evaluate whether CU (regular-users, n = 35 vs. non-using controls, n = 40) was associated with network topology metric differences after controlling for past-year alcohol use, age, sex, and cotinine levels; moderation by sex was also investigated. Regressions were run within CU group to test for associations between cannabis use patterns (lifetime CU, age of CU initiation, and past-year CU) and network topology. Finally, a network-based statistic (NBS) approach was used to search for connectome subcomponents associated with CU group, CU*sex, and patterns of CU.

**Results:**

No significant association between CU groups and ICN topology was observed. Sex moderation was observed; within male cannabis users, higher past-year CU was associated with significantly higher frontoparietal and ventral attention network (VAN) efficiency. Within female cannabis users, higher past-year CU was associated with significantly lower Default Mode Network assortativity. Within individuals who initiated CU before the age of 17, males had lower assortativity in the VAN and Somatomotor network. NBS analyses indicated that connectivity strength within a primarily right-lateralized subnetwork distributed throughout the connectome was significantly and reliably associated with past-year CU).

**Conclusion:**

The present findings suggest that subtle differences in resting-state network topology associated with CU may persist after an extended period of abstinence in young adults, particularly males, especially those with heavier past-year use and those who initiated CU earlier in life. While further replication is required in larger samples, these findings suggest potential neuroimaging correlates underlying long-term changes in brain network topology associated with CU.

## 1 Introduction

Cannabis is one of the most extensively used psychoactive drugs, with peak usage occurring during adolescence and young adulthood (1). Delta-9-tetrahydrocannabinol (delta-9-THC) is the primary psychoactive cannabinoid found in cannabis that is used for recreational purposes; delta-9-THC interacts with the endocannabinoid (eCB) system and acts as a partial agonist of the cannabinoid receptor 1 (CB1R) (2–4). CB1R are localized on neurons, astrocytes, microglia, and oligodendrocytes, and endogenous endocannabinoids (N-arachidonoylethanolamine, AEA; 2-arachidonoylglycerol, 2AG) are released in response to synaptic activity and regulate neurotransmitter release (5–8). The eCB system also contributes to numerous other processes linked with brain health, including cell differentiation, neurogenesis, energy metabolism, refinement of neuronal connections, pruning of dendrites, myelination of axons, as well as being involved in anti-inflammatory and immune functions (5–7, 9–14). Animal and human research has shown high density of CB1R and eCB signaling in the prefrontal cortex (PFC), parietal, cerebellar, limbic system, and ventral striatal regions (3, 4, 8, 15).

The eCB system demonstrates dynamic adolescent changes, with ligands (AEA, 2AG) increasing and CB1R expression peaking during the teenage years (15–20). These co-occur with other adolescent neurodevelopmental changes; subcortical regions (i.e., amygdala, hippocampus, nucleus accumbens) develop first in preadolescence and remain stable or slightly increase during later teen years (evidenced through volumetric and diffusion MRI investigations), while pruning of cortical (especially PFC and parietal cortices) gray matter and improved white matter integrity continues into early adulthood (21–29). These dynamic changes in the eCB system are hypothesized to at least partially underlie these adolescent neurodevelopmental processes, including changes in dendritic structure, synaptic pruning, fine-tuning of functional coupling, and enhancing white matter quality, particularly in the PFC, parietal, cerebellar, striatal, and limbic regions (3, 4, 8, 15, 30–32). Functional neuroimaging (fMRI) studies also demonstrate dynamic changes in the BOLD response during adolescence, with limbic and striatal response to salient and affective stimuli peaking resulting in increased “top-down” frontoparietal cognitive control and interhemispheric connectivity (25, 33–35). This has sparked interest in examining brain data using network-based statistics (i.e., “connectomes”) where specified regions are denoted as nodes and strength between nodes are evaluated (i.e., “edges”). These changes also correspond to a distributed reorganization of functional brain networks from “local” (i.e., within-network) to “distributed” (i.e., between-network) with increasing modularity, hierarchical organization, and greater specificity among within-network connections that mirror canonical adult resting-state networks (36–39).

Given the role of the eCB system during adolescent development, chronic exposure to cannabis may disrupt neuromaturation; there is evidence that repeated delta-9-THC may downregulate the eCB system (40–45) and alter glutamate-modulated synaptic refinement (46–49). Preclinical findings have suggested that repeated delta-9-THC dosing alters glucose uptake, dopamine, glutamate and eCB signaling, gene expression, microglial apoptosis, neuroplasticity, white matter quality, and functional coupling (50–56). However, the effects of delta-9-THC on distributed resting-state cortical networks in non-human animals, especially among adolescents and young adults, have not been well characterized.

### 1.1 Cannabis and human brain networks

Human longitudinal studies have collectively shown that chronic cannabis use (CU) during adolescence leads to modest cognitive decline, especially in the areas of executive functioning and verbal memory [for reviews see (57, 58)]. These cannabis-related deficits in cognitive function often correspond to differences in functional brain activity when both are assessed; for example, a recent meta-analysis focused on brain functional connectivity outcomes associated with CU found evidence for relatively widespread increased connectivity strength in late adolescent and adult (16–42 years old) cannabis users relative to controls, particularly in frontal-frontal, fronto-striatal, and fronto-temporal region pairings (59). Similarly, Hammond and colleagues conducted a meta-regression analysis on 1,216 cannabis using and 1,486 non-using adolescents and concluded that cannabis using youth demonstrate greater activation in rostral medial, ventral PFC, and ACC regions during executive control tasks, but blunted dorsal medial PFC and dorsal ACC response during affective processing tasks (60). Further, they found that extent of CUD, length of abstinence, as well as sex, were related to BOLD response in executive control, suggesting that severity of CU may be key indicators of neurobiological impacts of CU (60). Increased local connectivity in frontal and midbrain regions was also higher in individuals who met the criteria for CUD (*n* = 22) than non-using controls (*n* = 20), suggesting the pattern of hyperconnectivity may extend beyond cortical structures (61). However, not all seed-based analyses have indicated increased connectivity associated with CU.

Most studies highlighted in these reviews have utilized BOLD-response differences or seed-based functional connectivity analyses that focus solely on connectivity to a singular region, such as the anterior cingulate cortex (ACC) or insula. However, network-level patterns have emerged from these findings as many of the brain regions fall within the same set of brain networks: the Dorsal Attention Network (DAN; a set of brain regions associated with top-down, goal-directed processing of attention), the Ventral Attention Network (VAN; a set of brain regions associated with bottom-up, stimulus-driven processing of attention), the Frontoparietal Control Network [FPCN; a network commonly associated with executive function; (62)], and the DMN [a network commonly associated with self-referential thought; (63, 64)]. Evidence has been mixed regarding the links between adolescent and young adult CU and connectivity within the DMN. Some studies have found hypoactivity (65–67) while others indicate hyperactivity (68) among cannabis-using participants within the DMN using seed-based analyses. In terms of between-network connections, there is some evidence that hyperconnectivity between the DMN and VAN is associated with the acute feeling of being high (66). This association is further substantiated by the dose-dependent association between duration of CU and connectivity strength between key structures in the DMN and VAN (67). Harris et al. (69) identified decreased connectivity between the intraparietal sulcus (a key region in the DAN) and the insula (key region in the VAN) in abstinent cannabis users relative to healthy controls. Cannabis users have also demonstrated greater intra-network frontolimbic connectivity at rest compared to non-using youth (70). Ertl and colleagues (71) examined the impact of CU on connectivity in the frontoparietal control network (FPCN), they found greater seed-based functional connectivity in cannabis users vs. controls, though this association did not extend to other networks, suggesting some specificity of the effect. A data-driven approach supported this association, primarily indicating fronto-parietal brain regions as being hyper-active in heavy cannabis users (72). Therefore, to date, the FPCN, DMN, and DAN appear to be the most strongly associated with regular adolescent CU. Notably, these seed-based connectivity analyses typically do not quantify the degree in overlap between indicated brain regions and commonly found networks of brain regions which tend to coactivate with one another, or intrinsic connectivity networks (ICNs), as such, these results are difficult to place in a network context.

Given the complexity of cannabis effects on neuromodulation and glutamate-related functional coupling during development, the impact of CU may be better addressed by studying brain network topology. Analysis of brain activity on the level of network topology has the benefit of evaluating distributed functional brain activity using graph theory to represent the flow of information in the brain. This “network neuroscience” approach also reduces the issue of multiple comparisons in conventional fMRI analyses by allowing for analysis on multiple scales (e.g., network, module, or node level) and has proven to be informative at delineating specific network patterns and characteristics of interest within neuroscience investigations (73–76). Functional brain network characteristics have been reliably associated with dimensions of psychopathology, including substance use, and may provide a useful biomarker of adolescent neurodevelopment (35, 77–80). During adolescence, there is also evidence for age-associated increased integration of the VAN and FPCN with other specialized networks, suggesting that the developmental refinement of functional brain networks may not be uniformed in nature (81). Further, exposure to exogenous cannabis may differentially disrupt specific networks.

Few studies to date have examined network topology in adolescent and young adult recreational cannabis users. Global efficiency (*E*_*glob*_) is a common network marker generally representing the global strength of connections, it is defined as the average of the inverse shortest path length and represents how quickly (in terms of number of steps) information can flow in a network. In a weighted graph, this is heavily influenced by edge strength (i.e., positive correlation strength between nodes or regions). Thus, this statistic leverages both edge strength values and the higher-level structure of the network. In one of the first studies using brain network topology in acutely-abstinent (~12 h, *n* = 18) adolescents with CU disorder (CUD), Nestor and colleagues (82) found that individuals with CUD had reliably higher *E*_*glob*_, demonstrating greater whole-brain connectivity strength, during a reward processing task than matched healthy controls (*n* = 18). Greater connectivity strength was positively associated with age of CU onset, supporting the idea that differential use trajectories may influence patterns of brain activity (82). Both of these findings were consistent across connectome thresholding levels, suggesting reliable and robust effects in adolescents with CUD. In another data-driven approach to identify differences in functional connectomes in a mixed sample of adolescents and adults (age 18–40), Ramaekers and colleagues (83) found hyperconnectivity across all major brain networks, but most prominently in the DMN, DAN, and VAN, in recently abstinent chronic cannabis users (~24 h, *n* = 14) compared to abstinent occasional users (~7 days, *n* = 12). Using a meta-analytic approach, Blest-Hopley and colleagues (84) identified hyperactivity within the FPCN and DMN structures in abstinent cannabis using (> = 25 days, *n* = 98) vs. non-using (*n* = 106) adolescents. Taken together, preliminary findings suggest general hyperconnectivity brain patterns in cannabis users, particularly among the FPCN, VAN, and DMN regions, with mixed findings within the DAN and DMN. Notably, most of these analyses have not tested for the impact of more nuanced CU patterns, such as dose-effects, age of regular use onset, length of abstinence, or sex of the user on differences in functional brain network activity (85).

Another potential reason underlying inconsistent findings are potential unique sex effects on cannabis during neurodevelopment. There are significant sex-at-birth differences in typical adolescent brain development, with girls typically reaching structural maturity in limbic, striatal, and frontoparietal regions earlier than boys and demonstrating distinct patterns of functional connectivity maturation (29, 86–88). For example, males demonstrate progressively increases in putamen integration (in terms of participation coefficient) with age, but not females (89). On balance, animal studies have reported significant sex-variations in the eCB system, which plays a role in sexual dimorphism, including differences in CB1R receptor sensitivity, hormone-linked fluctuations in eCB signaling, and cannabis-related effects such as gene expression, brain morphometry, and neuroinflammation (50, 51, 56, 90–96). Human studies have also reported differential cognitive effects, with several studies finding increased male vulnerability (97–100) and some finding female vulnerability (97, 99, 101, 102). fMRI connectivity studies have also reported sex differences in the impact of cannabis on brain connectivity (50). However, not all analyses have indicated an interaction between cannabis use and sex (61, 65), and more studies examining sex differences are needed (59). Importantly, no studies to date have examined potential sex-differences in cannabis effects on network topology.

Prior literature has found that regular cannabis use can result in cognitive deficits and hyperconnectivity between brain areas, particularly ICNs associated with attentional processing and executive function, like the DAN, VAN, and FPCN (59, 83). These alterations in brain functioning may be due to the impact chronic cannabis use has on the eCB system via alterations in CB1 receptor density. Importantly, the eCB system is dynamic throughout the lifespan and is sexually dimorphic, which may explain sex differences in cannabis use outcomes. Very few studies have evaluated whether alterations in functional brain activity remain in regular users after prolonged abstinence. The current study aimed to examine markers of network connectivity in regular cannabis using adolescents and young adults after they completed a monitored three-week period of abstinence. Given the critical developmental time-period, we also sought to evaluate the hierarchical organization of brain networks as a marker of functional brain network maturation. Prior network-based analyses have quantified network organization through metrics like modularity, integration, and participation coefficient, all of which evaluate the degree to which subnetworks or modules are connected with one-another (82, 89). However, these metrics rely on one of many algorithms which allocate nodes into discrete modules (e.g., Louvain, Newman, etc.), resulting in a high number of researcher degrees of freedom. Alternatively, assortativity refers to the degree to which nodes are more likely to be neighbors with similar nodes (in terms of network characteristics like degree or clustering coefficient) and represents a metric of hierarchical organization free of any bias which might be introduced by module assignment. Assortativity can also be considered a metric of network robustness and resilience to damage (103). Thus, for our analyses we focused on both *E*_*glob*_and assortativity. We hypothesized that cannabis users would demonstrate significantly higher *E*_*glob*_ and lower assortativity. Further, we hypothesized that male users would demonstrate more robust relationships between CU and network topology compared to female users. Finally, we will examine the relationships between CU characteristics (age of regular use onset, past-year and lifetime use, and length of abstinence) and global and specific ICNs network outcomes. Sex differences will also be explored. We hypothesized that early CU initiation will be linked with less assortative networks, reflecting impaired maturation of the hierarchical structure of functional brain networks. We also predict that greater CU, and more recent use will be linked with hyperconnectivity, particularly in brain networks associated with executive functioning and attentional processing (FPCN, DAN & VAN). We predict these findings will be more robust in male users.

## 2 Materials and methods

### 2.1 Participants

Ninety-four adolescents and young adults (42 female) were recruited through local advertising for the larger parent study (PI: Lisdahl, R01 DA030354). Inclusion criteria included being between the ages of 16 and 26 years old, being a fluent English speaker, and willingness to abstain from all alcohol or drug use (except nicotine) for three weeks. Exclusion criteria included being left-handed, major neurological and metabolic disorders, past-year co-morbid independent Axis-I mood, anxiety, attentional, and psychotic disorders, prenatal medical issues or premature gestation <35 weeks, prenatal alcohol (>4 drinks/day or >7 drinks/week) or illicit drugs (>10 uses) exposure (by parent or youth report), inability to complete VO2 maximum testing, being categorized as a “heavy drinker” (Cahalan Criteria), and excessive illicit drug use in lifetime (>50 uses of any drug category except nicotine, alcohol, or cannabis). Abstinence from all alcohol and drug use were confirmed through self-report and drug toxicology. Eleven participants (3 female) did not have sufficient neuroimaging data available and were excluded from the current analyses. For the purposes of between group comparisons, regular cannabis users (CAN) were defined as individuals who had engaged in at least 44 cannabis uses in the past-year and at least 100 lifetime cannabis uses. Control participants were defined as individuals who had engaged in no more than 5 past-year cannabis uses and no more than 20 lifetime cannabis uses (104). Other substance use (>50 lifetime occasions) was exclusionary in the current study. Eight participants (4 female) were excluded from group-comparison analyses because they fell outside of the criteria for being CAN or control subjects, resulting in a sample size of 75 for comparisons between CAN (*n* = 35) and control subjects (*n* = 40). For evaluation of the impact of cannabis use characteristics within cannabis users (characterized as individuals who had abstained for greater than 20 days and less than 100 days), 7 subjects were excluded due to missing data in critical covariates, resulting in a sample size 39 cannabis users. Of note, participants are comprised of illicit cannabis use due to lack of legal recreational or medical laws surrounding cannabis use in Wisconsin, USA (data was collected prior to the 2018 Farm Bill).

### 2.2 Procedure

All study methods were approved by the UWM Institutional Review Board. Participants called in to a study line listed on the fliers posted around the community and local newspapers that highlighted a study recruiting active and sedentary participants with varying CU histories. Participant verbal consent, or verbal assent from minors, along with parent/guardian consent were obtained during a brief phone screen to assess for initial eligibility (i.e., age, MRI contraindications, and yes/no questions regarding psychiatric and substance use history). If the initial eligibility requirements were met, written consent/assent was received via mail and a 45 min, more detailed screening session was scheduled with both parents and youth. During this second session, more detailed demographic and medical history, physical health (to complete VO2 maximum testing), lifetime substance use [Customary Drinking and Drug Use Record; CDDR; (105, 106)] and psychiatric history (youth and parent versions of the Mini International Psychiatric Interview (MINI) or MINI-Kid if under 18 (107) were assessed. All participants and their parents were compensated $20 for the second screening session. Eligible participants were scheduled for study sessions. Ineligible participants were not informed of the specific reason for their exclusion to protect study integrity. Eligible youth completed five study sessions across 3.5–4 weeks [once a week for 3 weeks for the initial monitored abstinence period along with a mini-psychiatric and neuropsychological battery; see (108) for details]; during session 4, youth completed a detailed substance use interview, neuropsychological testing, and VO2 maximum testing. The fifth session was scheduled within 24–48 after session 4 and consisted of magnetic resonance imaging (MRI) scanning, TLFB update, and drug toxicology testing.

### 2.3 Measures

#### Substance Use Patterns.

The CDDR (105) was used to measure lifetime CU episodes, age of regular CU initiation (defined as weekly), and symptoms of CUD. The Timeline Follow Back (TLFB) calendar interview was used to measure past-year substance use according to standard units [alcohol (standard drinks), nicotine (number of cigarettes, hits of chew/snuff/pipe/cigar/hookah), cannabis (smoked/vaped flower, concentrates, and edibles were measured and dosing was converted to joints based grams)], other drugs including ecstasy/MDMA (tablets), cocaine (grams), methamphetamine (mg), prescription sedatives (pills), prescription stimulants (pills), hallucinogens (hits), GHB (occasions), ketamine (occasions), heroin or opium (hits) and inhalants (hits). Other substance use was primarily utilized for exclusion criteria for the current sample. Total past-year cannabis use (in grams), lifetime cannabis use episodes, age of regular cannabis use onset (weekly), and length of abstinence (in days) were used in the current analyses. Past-year alcohol use (standard units) was used as a covariate.

#### Drug Toxicology.

At each study session, drug toxicology urinalysis was completed screening for recent delta-9-THC, THCCOOH (delta-9-THC metabolite), amphetamines, barbiturates, benzodiazepines, cocaine, ecstasy/MDMA, methadone, methamphetamine, opiates, PCP (ACCUTEST SplitCup 10 Panel drug test) and cotinine concentrations (NicAlert). Cotinine was considered the most relevant covariate as a representation of acute nicotine use which has been known to influence BOLD signal. Past year nicotine use was not included in the modeling approach to prevent issues of multicollinearity. Participants were also administered the continuous sweat toxicology patch (PharmChek Drugs of Abuse Patch; PharmChem Inc., Fort Worth, TX, United States), which continuously monitored sweat toxicology and provided quantified values of THCCOOH (along with other drugs of abuse). Youth also completed breathalyzer screens for recent alcohol use at each session (Alco-Sensor IV; Inoximeters Inc., St. Louis, MO). If youth demonstrated increasing THCCOOH levels, indicating recent use, or new THCCOOH positive tests, they were offered up to one week extension to complete the monitored abstinence period; 95% of the youth completed the monitored abstinence period as scheduled. Cotinine level (day 5) was used as a covariate.

### 2.4 MRI data acquisition

High-resolution anatomical images were collected using a T1-weighted (T1w) spoiled gradient-recalled at steady-state pulse sequence (TR = 8.2 ms, TE = 3.4 s, TI = 450, and flip angle of 12°). The in-plane resolution of the anatomical images was 256 × 256 with a square field of view (FOV) of 240 mm. One hundred fifty slices were acquired at 1 mm thickness. Echo planar images (EPI) were acquired for eight minutes during eyes-closed rest using T2 × weighted gradient-echo EPI pulse sequence (TR = 2000 ms, TE = 25 ms, FOV = 240 mm, matrix 64 × 64 voxels, slice thickness = 3.7 mm., flip angle = 77 degrees, 40 contiguous axial slices) with 240 TRs of volume data acquired per run.

### 2.5 MRI preprocessing

Full MRI/fMRI preprocessing and functional connectome generation details can be found in the supplementary materials. Results included in this manuscript come from preprocessing performed using fMRIPrep 22.1.1 (109, 110); (RRID: SCR_016216), which is based on Nipype 1.8.5 (111, 112); (RRID: SCR_002502). Correction and registration was conducted using ANTs 2.3.3 (113), (RRID:SCR_004757) and images were normalized into standard space (MNI152NLin6Asym); (114). Functional images were skull-stripped, corrected for motion, and co-registered with the T1w-reference map Automatic removal of motion artifacts using independent component analysis (ICA-AROMA), (115) was performed on the preprocessed BOLD time-series after removal of non-steady state volumes and spatial smoothing with an isotropic, Gaussian kernel of 6 mm FWHM (full-width half-maximum). Corresponding “non-aggressively” denoised runs were then censored to exclude volumes using the “basic scrubbing” procedure outlined in Parkes et al. (116) such that all volumes with FDPower > 0.2 mm or BOLD data variance (DVARS) >3% were excluded from subsequent analyses (117). After scrubbing, only one subject was excluded for having less than 4 min of viable data. The resulting smoothed and scrubbed data were used to generate functional connectomes.

### 2.6 Functional connectome generation

The preprocessed, non-aggressively smoothed functional data was then parcellated using the NiftiLabelsMasker function in NiLearn v0.9.0 (171); (RRID:SCR_001362), using the 200 parcel 7 network atlas generated by Schaefer et al. (172). The parcel-wise mean BOLD signal was then subtracted from the values in the timeseries to center BOLD values for each parcel at a mean of 0. After this, every parcel was correlated with one another using the Pearson correlation to establish functional connectivity matrices for each session. The unique values from this matrix (i.e., the lower or upper diagonal) were extracted and the negative correlation values were set to zero. The resulting matrix was then normalized using a Fisher-*z* transformation and thresholded using estimated proportions of non-spurious correlations of .01, .1, .2, .35, and .5 in order to test the replicability of any findings across thresholds of network sparseness as typically used with network topology research (82, 118–120). While this approach inflates the number of comparisons, it also allows for greater reliability and comparability with other network-based analyses (36, 39, 81, 82). The resulting thresholded matrices were then treated as network objects in the form of weighted adjacency matrices.

### 2.7 Statistical analyses

#### Network-Based Statistics.

To examine whether CU group status, age of initiation and CU patterns (past-year, lifetime use) impacted subnetwork connectivity across all connectomes and thresholding levels, we utilized the Network-Based Statistic (NBS) approach across the entire sample of available neuroimaging data (*n* = 83). This identifies links between CU markers and connectivity patterns including potentially unique subnetworks, while leveraging the network structure of connected sets of edges and controlling for the high family-wise error (FWE) [see Levakov et al. (121) for continuous NBS analyses]. Edges were thresholded based on the degree of correlation between edge strength and the CU markers of interest. Multiple thresholds (.3, .325, .35, and .375) were evalueated to ensure the robustness of results.

#### Network Topology.

Global efficiency (*E*_*glob*_) and weighted assortativity (122) were calculated for each functional connectome on a whole-brain level and on the level of each of the 7 Yeo ICNs (123) using brainconn V.0.0.2 in Python 3.10.11 across all thresholding levels. Next, we conducted and exploratory analysis of the associations between cannabis group and use characteristics and brain network outcomes (*E*_*glob*_ and assortativity) using multiple linear regression within the “stats” package in R 4.2.1 (124). All models included age, sex, past-year alcohol use, and session 5 cotinine concentrations as covariates. Separate models were created for each brain network characteristic on the whole brain level and on the level of each of the 7 ICNs. The first set of models included group membership (regular cannabis users vs. controls) as a predictor (*n* = 64, 32 female). The second set of models only included participants who endorsed CU (*n* = 31, 11 female), and included age, sex, past-year alcohol use, length of cannabis abstinence, age of regular CU onset (dummy coded variable indicating use was initiated at or prior to age 17), past-year CU, and interaction terms for sex*past-year use, sex*early CU initiation, and past-year use*early CU initiation to examine interaction effects within the same models. This resulted in a total of 80 regression models. Results for all loadings from regression analyses include uncorrected *p*-values as well as *p*-values corrected for false discovery rate (FDR; *p*_*adj*_) within each thresholding level for the regressors of interest (heavy CU group and early initiation binary variables, past year CU, and interaction terms).

## 3 Results

### 3.1 Demographics

Table 1 shows the demographic distributions for all groups of participants. CU groups and age of initiation groups did not significantly differ in terms of age, sex, ethnic or racial identity and there were no significant correlations between any demographic variable and past-year CU, lifetime CU, or length of abstinence. Heavy cannabis users consumed more alcohol in the past-year and had higher cotinine levels than healthy control subjects; these were included as covariates. Neither framewise displacement nor number of volumes scrubbed were significantly associated with cannabis use characteristics or group membership.

### 3.2 Functional connectivity results

#### Network-Based Statistics: Cannabis Group Status.

Across all thresholds, group-wise NBS analyses were unable to identify subnetworks which were significantly different between cannabis users and controls, nor between cannabis users who initiated CU early and those who initiated CU later in life.

#### Network-Based Statistics: Cannabis Use Patterns.

**FIGURE 1:**
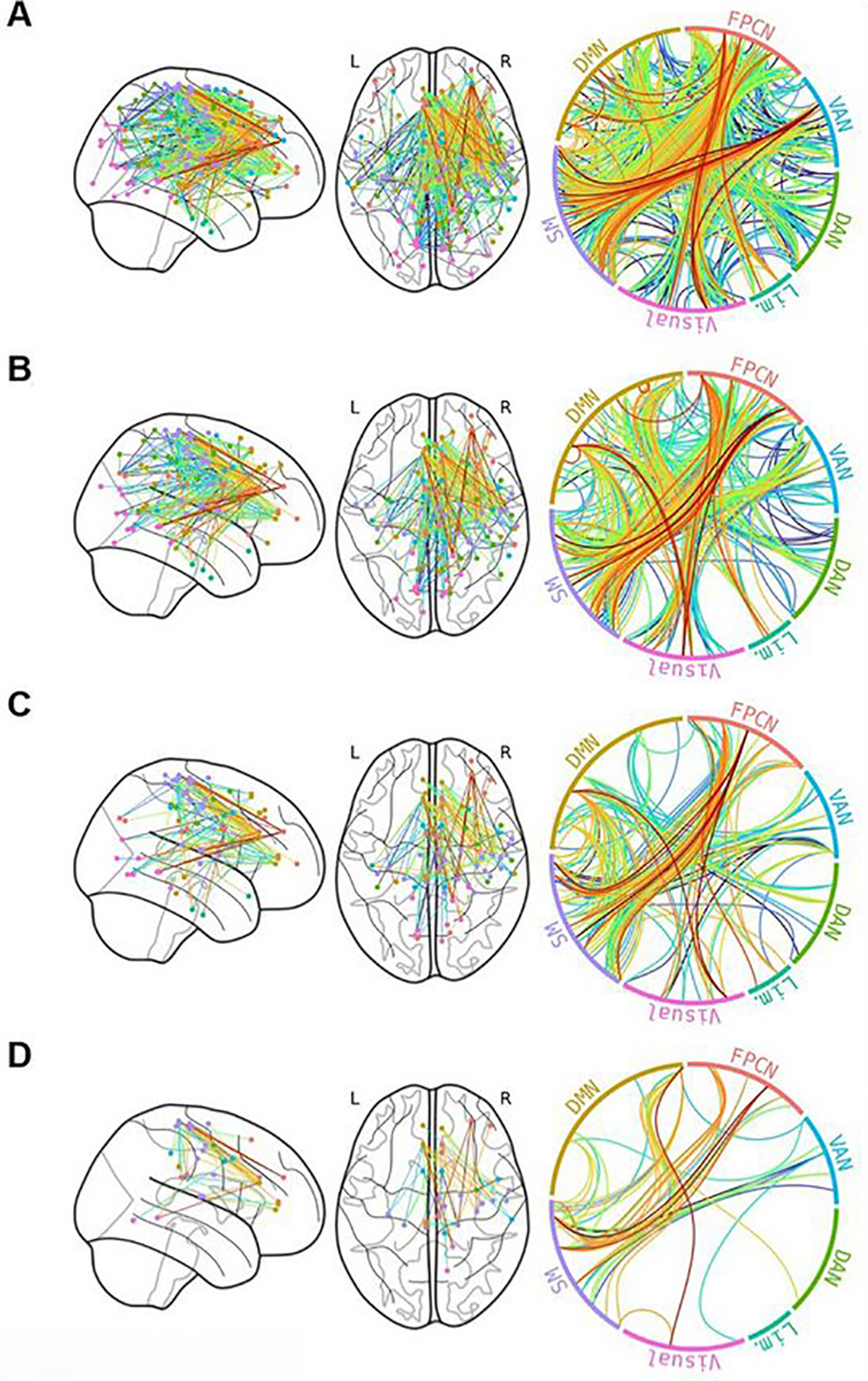
Network-based statistic subnetwork associated with past-year cannabis use among 79 adolescents at Pearson’s r thresholds of 0.3 (**A**), 0.325 (**B**), 0.35 (**C**), and 3.75 (**D**) edge color corresponds to the correlation between edge strength and past-year cannabis use. Connections appear to be primarily right-lateralized and between rather than within networks. Frontoparietal Control Network (FPCN), Ventral Attention Network (VAN), Dorsal Attention Network (DAN), Limbic Network (Lim.), Somatomotor Network (SM), Default Mode Network (DMN).

The continuous NBS approach (121) analysis found that increased **past-year CU** was positively associated with a subnetwork of edges at multiple threshold-levels (thresholds of .3, .325, .35, and .375; all *p*’s < .05); this subnetwork was comprised of brain regions from each of the ICN’s, and was primarily comprised of between-network edges from the FPCN, DMN, Somatomotor Network, and VAN (Figure 1). Nodes in the ACC, Frontal Pole, and Precentral Gyrus were consistently identified across thresholds. No relationships were found with lifetime CU.

### 3.3 Network characteristics

#### Cannabis Group & Network Topology:

After controlling for age, sex, alcohol use and cotinine levels, CU group status was not significantly related to *E*_*glob*_ or assortativity in any of the ICNs or on the whole-brain level (all *p*’s > .05). This null finding was consistent across thresholding levels. Cannabis Group*Sex. There were no significant cannabis group-by-sex interactions in predicting global or ICN network outcomes at all thresholds.

#### Cannabis Use Characteristics & Network Topology:

Within cannabis users, a the most stringent threshold (.01), whole-brain assortativity was significantly associated with duration of cannabis abstinence and the interaction term between early regular CU and past-year CU [*F*(10,28) = 2.55, *p* = .02, *R*^2^_*Adj*_ = .29]. Specifically, shorter **duration of abstinence** was associated with increased whole-brain assortativity [*t*(28) = 2.41, *b* = .003, *p* = .02, *p*_*adj*_ = .48, *f*^2^ = .32] and within late regular CU onset individuals, **past-year CU** was associated with higher whole brain assortativity [*t*(28) = −2.52, *b* = −.15, *p* = .02, *p*_*adj*_ = .48,*f*^2^ = .23]. There was also an observed interaction between past-year CU and early regular CU initiation for whole-brain *E*_*glob*_ [*F*(10,28) = 2.33, *p* = .038, *R*^2^_*Adj*_ = .26; *t*(28) = −2.08, *b* = −.009, *p* = .05, *p*_*adj*_ = .89, *f*^2^ = .16]. *post-hoc* evaluation revealed non-significant findings. At less stringent thresholds, shorter **duration of abstinence** was associated with increased assortativity in the **VAN** [0.35: *F*(10, 28) = 2.26, *p* = .04, *R*^2^_*Adj*_ = .25, *t*(28) = 2.08, *b* = .003, *p* = .05, *p*_*adj*_ = .67, *f*^2^ = .28; 0.5: *F*[10, 28] = 2.32, *p* = .04, *R*^2^_*Adj*_ = .26, *t*(28) = 2.05, *b* = .003, *p* = .05, *p*_*adj*_ = .80, *f*^2^ = .29]. Greater **past-year use** was also associated with decreased *E*_*glob*_ in the **FPCN** at the threshold of.35 [*F*(10, 28) = 2.59, *p* = .02, *R*^2^_*Adj*_ = .3, *t*(28) = −2.22, β = −.08, *p* = .03, *p*_*adj*_ = .54, *f*^2^ = .11]. Effect size ranges for these findings were generally small to medium (*f*^2^ range .11–.32).

#### Cannabis Use Characteristics*Sex & Network Topology:

Within cannabis users, there were significant interactions between **sex** and **past-year CU** in association with *E*_*glob*_ in the **FPCN** at thresholds of .35 [*F*(10,28) = 2.59, *p* = .02, *R*^2^_*Adj*_ = .3; *t*(28) = 3.16, *b* = −.13, *p* = .004, *p*_*adj*_ = .18, *f*^2^ = .37] and .5 [*F*(10,28) = 3.29, *p* = .006, *b* = −.13, *R*^2^_*Adj*_ = .38; *t*(28) = 3.26, *p* = .003, *p*_*adj*_ = .14, *f*^2^ = .41]. Within **males**, past-year cannabis use was positively associated with *E*_*glob*_ at both thresholds [.35: *F*(7, 18) = 3.13, *p* = .02, *R*^2^_*Adj*_ = .37; *t*(18) = 3.07, β = .06, *p* = .007, *f*^2^ = .43;.5: *F*[7, 18] = 4.79, *p* = .003, *R*^2^_*Adj*_ = .51; *t*(18) = 4.24, β = .07, *p* < .001, *f*^2^ = .74], while there was no significant association within females. Similarly, there were significant interactions between sex and past-year cannabis use on assortativity within the **Somatomotor** network at thresholds of .2 [*F*(10,28) = 3.37, *p* = .005, *R*^2^_*Adj*_ = .38; *t*(28) = 2.38, *b* = .136, *p* = .02, *p*_*adj*_ = .58, *f*^2^ = .21] and .35 [*F*(10,28) = 3.29, *p* = .006, *R*^2^_*Adj*_ = .38; *t*(28) = 2.94, *b* = .16, *p* = .007, *p*_*adj*_ = .31, *f*^2^ = .34]; within **males** only, increased pass year cannabis use was associated with increased Somatomotor assortativity [.2: *F*(7, 18) = 5.79, *p* = .001, *R*^2^_*Adj*_ = .57; *t*(18) = 4.32, β = .1, *p* < .001, *f*^2^ = 1.29;.5: *F*(7, 18) = 4.08, *p* = .007, *R*^2^_*Adj*_ = .46; *t*(18) = 4.3, β = .1, *p* < .001, *f*^2^ = .99]. Effect size ranges for these findings were medium to large (*f*^2^ range .21–1.29).

Interaction terms between **sex** and early **regular CU onset** accounted for a significant portion of variance in assortativity in the **Somatomotor** network [1: *F*(10,28) = 2.49, *p* = .028, *R*^2^_*Adj*_ = .28; *t*(28) = 2.72, *b* = .31, *p* = .01, *p*_*adj*_ = .18, *f*^2^ = .24;.2: *F*(10,28) = 3.37, *p* = .005, *R*^2^_*Adj*_ = .38; *t*(28) = .2.75, *b* = .24, *p* = .01, *p*_*adj*_ = .49, *f*^2^ = .18] and **VAN** [35: *F*(10,28) = 2.26, *p* = .04, *R*^2^_*Adj*_ = .25; *t*(28) = 2.39, *b* = .29, *p* = .02, *p*_*adj*_ = .57, *f*^2^ = .21;.5: *F*(10,28) = 2.32, *p* = .04, *R*^2^_*Adj*_ = .26; *t*(28) = 2.83, *b* = .29, *p* = .009, *p*_*adj*_ = .41, *f*^2^ = .25]. For both networks, *post-hoc* regressions indicated that within **males** only, early regular CU onset was associated with decreased assortativity [Somatomotor.1: *F*(7, 18) = 2.99, *p* = .029, *R*^2^_*Adj*_ = .36; *t*(18) = −2.99, *b* = −.22, *p* = .008, *f*^2^ = .21; Somatomotor .2: *F*(7, 18) = 5.79, *p* = .001, *R*^2^_*Adj*_ = .57; *t*(18) = −3.69, *b* = −.17, *p* = .002, *f*^2^ = .18; VAN .35: *F*(7, 18) = 2.73, *p* = .04, *R*^2^_*Adj*_ = .33; *t*(18) = −2.47, *b* = −.19, *p* = .02, *f*^2^ = .13; VAN .5: *F*(7, 18) = 3.12, *p* = .02, *R*^2^_*Adj*_ = .37; *t*(18) = −2.93, *b* = −.18, *p* = .009, *f*^2^ = .27]. Effect size ranges for these findings spanned small to medium (*f*^2^ range .13–.27). A list of whole model statistics relevant to the results reported here can be found in Table 2 and a summary of significant findings can be found in Table 3.

## 4 Discussion

CU peaks during late adolescence into young adulthood, a period of significant ongoing neurodevelopment. Prior work has suggested subtle effects of chronic CU on functional connectivity during adolescence, especially in the VAN, FPCN, DMN, and DAN, though seed-based findings have been inconsistent across networks. Our study aimed to expand upon this work by examining the impact of regular CU on brain network connectivity, utilizing both network based statistics (NBS) and network topology approaches, in adolescents and young adult cannabis users after a period of three weeks of monitored abstinence. We also examined whether sex moderated these findings.

Using an NBS approach, we found that increased past-year CU was linked with increased subnetwork of edges at multiple thresholds (.3, .325, .35, .375), and these primarily included *between-network edges* from the VAN, DMN, Somatomotor Network, and FPCN. This finding is consistent with the general pattern observed in the network topology analysis, finding subtle relationships between CU patterns and network outcomes in the VAN, Somatomotor network, and FPCN, and less so in the DMN. The subnetwork consistently comprised primarily of right-hemisphere structures, representing the first evidenced lateralization of the impact of CU on brain connectivity. Though we could not examine sex differences in the NBS approach, the exploratory regression analyses demonstrated primary male vulnerability in the VAN, FPCN and Somatomotor networks. This hyperconnectivity, especially in the right hemisphere, may suggest cannabis-related subtle differences in sex-related hemispheric connectivity asymmetry during middle to late adolescence (125). These differences may underlie cannabis-related reduced cognitive performance in right-hemisphere associated tasks that require visuospatial and visuo-motor integration, including visuospatial function (126), psychomotor sequencing ability (98, 127–129), spatial working memory (127, 130–135), complex visuospatial attention (98, 129, 130, 135–141), and motor-based cognitive control (98, 132, 133, 137, 138, 140, 142–144). Further, hyperconnectivity between the VAN and the other networks could represent a higher-likelihood of switching between network states (145), a process which is known to be associated with cognitive task performance (146). On the other hand, the increased connectivity associated with past-year cannabis use might represent a more disorganized connectome as a result of dysregulation of CB1 receptor density. This is supported by the high representation of the ACC in the subnetwork of hyperconnectivity associated with past-year CU. The high density of CB1 receptors in the ACC may make it particularly prone to dysregulation through heavy cannabis use, which is supported by prior research that has demonstrated reduced rACC volume linked with reduced emotional discrimination (173), blunted BOLD response during fearful face processing (174), and hyperconnectivity between bilateral rACC, left amygdala and left insula at rest (70) in regular cannabis using adolescents and young adults. Thus, additional research is needed to specifically examine the impact of regular cannabis use on ACC development during the critical stage of adolescent development.

In an exploratory follow-up analyses, we also aimed to examine effects of more nuanced cannabis use characteristics and potential sex differences on brain network topology in the whole brain, VAN, DAN, FPCN, Somatosensory network and DMN in adolescents and young adults. Notably, due to the large number of statistical tests, none of the comparisons of interest survived FDR correction for multiple comparisons despite medium to large effect. Still, we believe the pattern of results, emphasizing threshold values and effect sizes, are worth reporting in order to support replication. We found that at the group level, CU was not significantly linked with network outcomes. However, more nuanced exploratory analyses found preliminary evidence that increased past-year CU was associated with greater *E*_*glob*_ in the FPCN (.35 threshold), though this was a small effect size (*f*^2^ = .11). Shorter abstinence from CU was associated with greater assortativity on the whole-brain level (.01 threshold, *f*^2^ = .32) and within the VAN (.35 and .5 thresholds, *f*^2^ = .28, .29). Further, in later onset cannabis users, increased past year use was linked with increased assortativity at the whole brain level (.01, *f*^2^ = .32). Preliminary sex-dependent findings were also revealed; increased past year CU was associated with *E*_*glob*_ in the FPCN in males only (.35 and .5, *f*^2^ = .43, .74). We also found links between increased past-year CU and greater assortativity in the Somatomotor network in males across two thresholds (.2, .35, *f*^2^ = .43, .74). Interestingly, we found that earlier regular CU onset was associated with decreased assortativity in the VAN (thresholds of.35 and .5; *f*^2^ = .13, .27) and Somatomotor network (thresholds of .1 and .2; *f*^2^ = .21, .18) in male cannabis users, suggesting reduced connectivity in this subset of male users. While the directionality of these effects were consistent across thresholding levels, the findings were not consistently significant across thresholding levels. This is in contrast to findings such as those found by Nestor et al. (82), which were global in nature and were consistent across thresholding levels. This differential finding may be due to characteristics of the sample, as their study was almost entirely comprised of male participants and exclusively included youth being treated for CUD following roughly 12 h of abstinence, while ours included a community sample of recreational users with a range of use (weekly to daily) and following a minimum of three weeks of monitored abstinence (with average >30 days).

Still, taken together, these preliminary findings generally support the main NBS findings demonstrating patterns of hyperconnectivity associated with increased severity of use, especially in male user and early onset users. The VAN underlies stimulus-driven attention processes, many of which are negatively associated with cannabis use (83, 147, 148). The NBS analyses in which significant associations between CU frequency and brain network activity were identified were primarily driven by past-year use, rather than lifetime use. This is fairly consistent with other attention based neurocognitive dose-dependent findings (67, 83, 99). Interestingly, in the exploratory analyses, at more lenient thresholds (.35 and .5) and when accounting for total past-year use and earlier age of onset, shorter periods of abstinence in this case were linked with *greater* assortativity in the VAN. Thus, acute recovery of function reported in other analyses (73, 108, 149–151) may not explain the reduced assortativity in the male early onset users observed here. Prior analyses have not strongly associated the Somatomotor network with CU, but not many analyses have tested for sex-dependent effects and included relatively small sample sizes. The increased VAN and FPCN connectivity strength and lower assortativity in the Somatomotor network in males with risky cannabis use patterns may suggest male vulnerability to cannabis-effects, especially in these frontal-parietal and temporal-frontal networks (99). These findings suggest that nuanced cannabis markers may be linked with whole brain, VAN, FPCN, and Somatomotor network connectivity strength and hierarchical structure and results were dependent upon dose of exposure, age of onset, and sex of the user. As stated above, results did not survive correction for multiple comparisons; given the low power of this subset of participants, additional studies are needed with larger sample sizes that include a range of cannabis characteristics in order to replicate findings. Additionally, future studies conducted in a large, sex-balanced sample utilizing repeated neuroimaging during a period of monitored abstinence are needed to further characterize these cannabis-related sex differences in network topology recovery of function.

The preliminary evidence of sex differences in associations between CU patterns and ICN global efficiency and assortativity may be due to sex differences in brain maturation rates. As males develop later than females, they may be particularly sensitive to heavy CU during middle adolescence in terms of functional frontoparietal, frontotemporal and Somatomotor development and synaptic refinement. The eCB system dynamically shifts in function throughout development, with some processes like presynaptic signal modulation from the PFC to the hippocampus and amygdala do not mature until late adolescence, especially in boys (152). CB1 receptor density tends to be at its highest *prior* to this shift (153). Early CU initiation may also interrupt the eCB system’s other functions (e.g., synaptic pruning & myelination) which may have more long-term impacts on the organizational structure of brain networks. The network specificity may represent a particular sensitivity of certain networks to eCB system modulation or may be due to sex differences in terms of which networks underwent this refinement during a person’s peak CU. The lower assortativity in early vs. late CU males in the Somatomotor network and the VAN may indicate that eCB functioning in these networks had already shifted from the more long-term organizational effects to more neuromodulatory effects. The result is that early CU males display less organized brain network structures, similar to what would be expected from younger individuals. However, it is important to note that the low number of female participants in the late CU initiation group may have prevented meaningful comparisons between females in the early and late CU initiation groups and between males and females who initiated CU later in life. Thus, longitudinal studies examining the relationships between endogenous eCB circulating levels (154), detailed cannabis use patterns, and brain network topology development are needed to further characterize the impact of escalating use on functional activity patterns across development in boys and girls.

These findings should be considered carefully in the context of our sample characteristics and potential limitations. Our participants were healthy young adults who were willing to abstain from CU for at least 2 weeks, limiting the generalizability to clinical populations, younger adolescents, or older adults. The resting-state scan was also only eight minutes which is lower than some recommend (155), but still long enough to attain replicability (156); still, longer scan time collection may result in more robust or reproducible findings. These analyses were cross-sectional in nature and cannot account for causality; though dose-dependent findings were reported, premorbid differences may predict more severe cannabis use trajectories. Prospective, longitudinal studies will need to replicate findings. Further, given the relatively small sample size of cannabis users and resulting low statistical power, future studies should expand the network-based analytical framework with a large sample of cannabis users with diverse use characteristics to evaluate the generalizability of these findings. The current sample participants had a range of abstinence; repeated, longitudinal MRI studies examining recovery of function over a more controlled range are needed. Because of the large shift in availability of cannabis products in recent years, more nuanced characterizations of CU (e.g., method of consumption, cannabinoid content, potency) will be necessary to address additional predictors of cannabis-related effects (157–160). Particular care should be taken to include female participants and evaluate sex differences, as females are typically underrepresented in CU research (161) and demonstrate differential cannabis use patterns. Future studies should further examine the impacts of sex steroid hormones, particularly estradiol, should also be considered given their interaction with CB1 receptors (92, 162, 163), eCB levels (164–166), and CU outcomes (167–170).

Primary results revealed that, as a group, healthy cannabis-using adolescents and young adults who had abstained from CU for at least 2 weeks did not differ from non-using youth. The most consistent finding was that increased past-year cannabis use was associated with a distributed pattern of hyperconnectivity, particularly with regions with high CB1 receptor density like the ACC, and exploratory analyses revealed that effects may also be dependent upon age of regular cannabis use onset length of cannabis abstinence, and sex of the user. However, network topology results did not emerge consistently across all thresholding levels evaluated and were no longer significant after FDR correction for multiple comparisons, suggesting a larger sample size is needed to replicate findings related to cannabis use characteristics. In male users, age of regular CU initiation appeared to be associated with hierarchical brain network structure, a potential index of functional brain development. This could indicate that CU during adolescence could have subtle impacts on brain function, especially in male early onset, heavy cannabis users. These findings reinforce the need to restrict access and discourage use among developing adolescents. Further, these findings highlight the critical need to attend to sex differences in the eCB system, its role in adolescent neurodevelopment, and CU outcomes. Large-scale longitudinal studies are needed to further characterize the impact of nuanced cannabis use patterns on brain development of functional networks across development. These findings add to the growing literature that cannabis-related effects are likely nuanced, complex, and require large samples with diverse cannabis characteristics in order to examine interactions between factors [see (85)].

## Supplementary Material

Data_1

The Supplementary Material for this article can be found online at: https://www.frontiersin.org/articles/10.3389/fradm.2025.1549771/full#supplementary-material

## Figures and Tables

**TABLE 1:** Demographics for cannabis use group and early (prior to age 17) and late initiation cannabis users.

Demographic variable		Cannabis users (*n* = 39)		
	Healthy controls	Early initiation cannabis users	Late initiation cannabis users	Heavy cannabis users
	(*n* = 40)	(*n* = 24)	(*n* = 15)	(*n* = 35)
	% or *M* (*SD*)	% or *M* (*SD*)	% or *M* (*SD*)	% or *M* (*SD*)
	Range	Range	Range	Range
Age (y)	21 (2.67)	21.08 (2.22)	21.93 (2.46)	21.46 (2.13)
	16–25	17–24	18–26	17–26
Reading score (WRAT-IV)	106.1 (9.81)	107.17 (13.42)	105.47 (9.08)	104.66 (13)
	87–133	80–133	90–126	72–133
Sex (% female)	55%	37.5%	27%	37.1%
% Caucasian	73%	63%	80%	60%
% Not Hispanic/Latino/a	90%	71%	93%	77%
Past-year Alcohol Use (Standard Drinks)*	108.77 (170.98)	299.52 (305.63)	297.32 (271.56)	325 (301.28)
	0–698.5	24–1,120.5	10–800	0–1,120.5
Cotinine level*	1.1 (0.71)	2.13 (1.92)	1.33 (1.45)	1.86 (1.9)
	0–3	0–6	0–6	0–6
Past-year Nicotine Use	0.56 (1.98)	230.45 (532.15)	66.2 (238.86)	189.78 (466.57)
	0–12	0–1,867	0–929	0–1,867
Past-year Cannabis Use (joints)*	0.39 (1.15)	391.80 (543.53)	185.69 (223.63)	429.66 (447.48)
	0–5	4–2,306	1–548.5	44.7–2,306
Lifetime Cannabis Use (joints)*^+^	2.44 (4.99)	1,375.67 (1,620.71)	487.4 (543.65)	1,200.74 (1,389.0)
	0–20	25–6,000	5–1,668	101–6,000
Age of regular cannabis use initiation (weekly)^+^	-	14.71 (1.23)	18.4 (1.35)	16 (2.17)
		12–16	17–21	12–21
Duration of Abstinence (Days)	-	35.12 (15.35)	31.4 (13.19)	31.31 (23.19)
		21–77	22–72	17–150

Differences between healthy controls and heavy cannabis users at *p* < .05 are denoted by (*). Differences between early and late initiators are denoted by (^+^).

**TABLE 2:** Whole model statistics for significant models in which CU characteristics within cannabis users accounted for a significant portion of variance in network topology metrics of interest.

Network anylized		Global efficiency				Assortativity			
	Threshold	*df*	*F*	*p*	*R* ^2^_*Adj*_	*df*	*F*	*p*	*R* ^2^_*Adj*_
**Whole Brain**									
	.01	(10, 28)	2.33	.037	.26	(10, 28)	2.54	.025	.29
**VAN**									
	.35					(10, 28)	2.26	.044	.25
	.5					(10, 28)	2.32	.039	.26
**FPCN**									
	.35	(10, 28)	2.59	.023	.30				
	.5	(10, 28)	3.29	.006	.38				
**Somatomotor**									
	.1					(10, 28)	2.49	.028	.28
	.2					(10, 28)	3.37	.005	.38
	.35					(10, 28)	3.29	.006	.38

**TABLE 3:** Cannabis Use Characteristics & Network Topology. Significant associations between brain network topology and cannabis use characteristics are summarized here.

CU characteristic	Whole-Brain	VAN	DAN	FPCN	Som.	DMN
↑ Past-year CU	-	-	-	↑ *E*_*glob*_ (.35)	-	-
↑ Past-year CU * Sex	-	↑ E_*glob*_ ♂ (.01)	-	↑ *E*_*glob*_ ♂ (.35, .5)	↑ Assort ♂ (.2, .35)	-
↓ CU Initiation Age	-	-	-	-	-	-
↓ CU Initiation Age * Sex	-	↓ Assort ♂ (.35, .5)	-	-	↓ Assort ♂ (.1, .2)	-
↓ Abstinence	↑ Assort (.01)	↑ Assort (.35, .5)	-	-	-	-

Thresholds are indicated in parentheses. Global Efficiency (*E*_*glob*_), Assortativity (Assort), cannabis use (CU), Positive relationship (↑), negative relationship (↓), male (♂), female (♀).

## Data Availability

The data analyzed in this study is subject to the following licenses/restrictions: The raw data supporting the conclusions of this article will be made available by the authors, without undue reservation. Requests to access these datasets should be directed to Krista M. Lisdahl, medinak@uwm.edu.
