# Supplementary material for: Network topology and cannabis use following two weeks of monitored abstinence: moderation of sex and patterns of use findings
Source: Front Adolesc Med. Author manuscript; Available in PMC 2026 Mar 12. (PMC12978186; doi:10.3389/fradm.2025.1549771)
Supplement: Data_1 [file NIHMS2152534-supplement-Data_1.pdf]

Supplementary Material

1 MRI Preprocessing

Results included in this manuscript come from preprocessing performed using fMRIPrep 22.1.1 (Esteban et al., 2019; Markiewicz et al., 2024; RRID:SCR_016216), which is based on Nipype 1.8.5 (Esteban et al., 2022; Gorgolewski et al., 2011; RRID:SCR_002502).

1.1 Anatomical data preprocessing

For each subject, there were at least 2 T1w images. Each image was corrected for intensity non-uniformity (INU) with N4BiasFieldCorrection (Tustison et al., 2010), distributed with ANTs 2.3.3 (Avants et al., 2008, RRID:SCR_004757). The T1w-reference was then skull-stripped with a Nipype implementation of the antsBrainExtraction.sh workflow (from ANTs), using OASIS30ANTs as target template. Brain tissue segmentation of cerebrospinal fluid (CSF), white-matter (WM) and gray-matter (GM) was performed on the brain-extracted T1w using fast (FSL 6.0.5.1:57b01774, RRID:SCR_002823, Zhang et al., 2001). A T1w-reference map was computed after registration of the T1w images (after INU-correction) using mri_robust_template (FreeSurfer 7.2.0, Reuter et al., 2010). Volume-based spatial normalization to two standard spaces (MNI152NLin2009cAsym, MNI152NLin6Asym) was performed through nonlinear registration with antsRegistration (ANTs 2.3.3), using brain-extracted versions of both T1w reference and the T1w template. The following templates were selected for spatial normalization: ICBM 152 Nonlinear Asymmetrical template version 2009c (Fonov et al., 2009), RRID:SCR_008796; TemplateFlow ID: MNI152NLin2009cAsym], FSL's MNI ICBM 152 non-linear 6th Generation Asymmetric Average Brain Stereotaxic Registration Model (Evans et al., 2012); RRID:SCR_002823; TemplateFlow ID: MNI152NLin6Asym).

1.2 Functional data preprocessing

For each functional MRI (fMRI) scan, the following preprocessing steps were performed. First, a reference volume and its skull-stripped version were generated using a custom methodology of fMRIPrep. Head-motion parameters with respect to the BOLD reference (transformation matrices, and six corresponding rotation and translation parameters) are estimated before any spatiotemporal filtering using mcflirt (FSL 6.0.5.1:57b01774, Jenkinson et al., 2002). The BOLD time-series (including slice-timing correction when applied) were resampled onto their original, native space by applying the transformations to correct for head-motion. These resampled BOLD time-series will be referred to as preprocessed BOLD in original space, or just preprocessed BOLD. The BOLD reference was then co-registered to the T1w reference using mri_coreg (FreeSurfer) followed by flirt (FSL 6.0.5.1:57b01774, Jenkinson and Smith, 2001) with the boundary-based registration (Greve and Fischl 2009) cost-function. Co-registration was configured with nine degrees of freedom to account for distortions remaining in the BOLD reference.

Several confounding time-series were calculated based on the preprocessed BOLD: framewise displacement (FD), DVARS and three region-wise global signals. FD was computed using two formulations following Power [absolute sum of relative motions, Power et al. (2014)] and Jenkinson [relative root mean square displacement between affines, Jenkinson et al. (2002)]. FD and DVARS are calculated for each functional run, both using their implementations in Nipype [following the definitions by Power et al., 2014)]. Frames that exceeded a threshold of 0.5 mm FD or 1.5 standardized DVARS were annotated as motion outliers.

The BOLD time-series were resampled into standard space, generating a preprocessed BOLD run in MNI152NLin2009cAsym space. First, a reference volume and its skull-stripped version were generated using a custom methodology of fMRIPrep. Automatic removal of motion artifacts using independent component analysis (ICA-AROMA, Pruim et al., 2015) was performed on the preprocessed BOLD on MNI space time-series after removal of non-steady state volumes and spatial smoothing with an isotropic, Gaussian kernel of 6mm FWHM (full-width half-maximum). Corresponding “non-aggressively” denoised runs were produced after such smoothing. Additionally, the “aggressive” noise-regressors were collected and placed in the corresponding confounds file. All resamplings can be performed with a single interpolation step by composing all the pertinent transformations (i.e. head-motion transform matrices, susceptibility distortion correction when available, and co-registrations to anatomical and output spaces). Gridded (volumetric) resamplings were performed using antsApplyTransforms (ANTs), configured with Lanczos interpolation to minimize the smoothing effects of other kernels (Lanczos, 1964). Non-gridded (surface) resamplings were performed using mri_vol2surf (FreeSurfer).

2 Supplementary Tables

Table S1.

	Non-Cannabis Users (n = 37)		Cannabis Users (n = 39)	
	Male<br>(n = 15)<br>% or *M* (*SD*)<br>Range	Female<br>(n = 22)<br>% or *M* (*SD*)<br>Range	Male<br>(n = 26)<br>% or *M* (*SD*)<br>Range	Female<br>(n = 13)<br>% or *M* (*SD*)<br>Range
Age (y)	21.07 (3.10)	21.09 (2.49)	21.04 (2.31)	22.15 (2.27)
Reading score (WRAT-IV)	107.20 (9.06)	104.64 (10.68)	107.77 (13.13)	102.15 (7.72)
% Caucasian	67.67%	72.73%	80.77%	46.15%
% Not Hispanic/Latino/a	93.33%	81.82%	80.77%	76.92%
Past-year Alcohol Use (Standard Drinks)	131.37 (196.27)	68.43 (97.89)	312.46 (294.96)	271.12 (287.39)
Cotinine level	1.07 (.70)	1.18 (.73)	2.12 (2.08)	1.23 (.60)
Past-year Nicotine Use	.20 (.41)	.80 (2.64)	234.87 (534.73)	32.10 (68.48)
Past-year Cannabis Use (joints)	.27 (.70)	.05 (.21)	364.40 (520.38)	208.79 (274.96)
Lifetime Cannabis Use (joints)	1.60 (5.14)	2.52 (5.11)	1164.42 (1621.71)	773.23 (629.86)
Age of regular cannabis use initiation (weekly)			16.27 (2.20)	15.85 (2.30)
Duration of Abstinence (Days)			35.12 (16.41)	30.85 (9.56)

Population comparison of Male and Female cannabis users and non-users. The 39 cannabis users does not include 7 cannabis users within missing data which were withheld from the analysis within cannabis users.
